# From dysbiosis to precision therapy: decoding the gut-bladder axis in bladder carcinogenesis

**DOI:** 10.3389/fonc.2025.1630726

**Published:** 2025-07-10

**Authors:** Ze-qiang Liu, Xiao-ying Yang, Jia-hong Chen, Si-cheng Ge, Shi-xue Dai, Sheng-huang Zhu, Zhi-yong Xian

**Affiliations:** ^1^ The Second School of Clinical Medicine, Southern Medical University, Guangzhou, Guangdong, China; ^2^ The First School of Clinical Medicine, Gannan Medical University, Ganzhou, Jiangxi, China; ^3^ Department of Urology, Cancer Center, National Regional Medical Center, Guangdong Provincial People’s Hospital Ganzhou Hospital, Ganzhou, Jiangxi, China; ^4^ Department of Gastroenterology, Guangdong Provincial Geriatrics Institute, National Key Clinical Specialty, Guangdong Provincial People’s Hospital, Guangdong Academy of Medical Sciences, Southern Medical University, Guangzhou, Guangdong, China; ^5^ Department of Gastroenterology, Geriatric Center, National Regional Medical Center, Guangdong Provincial People’s Hospital Ganzhou Hospital, Ganzhou, Jiangxi, China; ^6^ Department of Urology, Guangdong Provincial People’s Hospital, Guangdong Academy of Medical Sciences, Southern Medical University, Guangzhou, Guangdong, China

**Keywords:** gut-bladder axis, bladder cancer, microbiota, dysbiosis, probiotics

## Abstract

The gut-bladder axis (GBA), a bidirectional network connecting gastrointestinal and urinary systems, has recently emerged as a pivotal focus in bladder cancer research. Beyond conventional risk factors, gut dysbiosis, aberrant microbial metabolites, and neuro-immune pathway disruptions have been implicated in tumorigenesis and progression. Short-chain fatty acids (SCFAs), microbial-derived metabolites, are shown to indirectly modulate tumor behavior through immune microenvironment regulation and inflammatory response attenuation. Cross-organ crosstalk is further mediated by neural pathways (e.g., vagal signaling) and shared receptors, including the Farnesoid X Receptor (FXR) and Toll-like Receptor 4 (TLR4). Novel therapies leveraging microbial ecology principles demonstrate potential, including immune checkpoint inhibitors combined with microbiota modulation (e.g., *Parabacteroides distasonis*-enhanced PD-1 efficacy), probiotics to reverse chemoresistance, and microbiota reprogramming for SCFA-targeted strategies. However, molecular mechanisms underlying GBA-host interactions remain poorly characterized. Clinical translation is hindered by limited cohort sizes and interindividual heterogeneity. Current studies, while revealing partial pathways, face methodological inconsistencies, particularly in urinary microbiome profiling, and a lack of longitudinal human data. Future breakthroughs will require multi-omics integration, organoid-based models, and interdisciplinary collaboration to address these gaps.

## 1 Introduction

Bladder cancer (BC) is ranked among the top ten most prevalent malignancies worldwide. According to WHO projections, approximately 1.2 million new cases are expected to be diagnosed globally by 2040 (1). Most BC cases originate from urothelial precursor lesions, which evolve through two distinct pathways termed papillary and non-papillary trajectories (2, 3). Approximately 80% of urothelial carcinomas manifest as superficial papillary tumors. While these neoplasms generally lack bladder wall invasion, high recurrence rates are typically observed following local treatment (3). The remaining 20% comprise non-papillary carcinomas, in which a high propensity for aggressive growth is exhibited (4–6).

While traditional risk factors for bladder cancer (e.g., smoking, arsenic exposure) have been extensively studied (6–9), recent evidence suggests dysregulation of the gut-bladder axis (GBA) may represent a novel pathogenic mechanism (9). The GBA, defined as a bidirectional network linking the gut and bladder via microbial communities, metabolites, immune signaling, and neural pathways (10, 11), operates via three core mechanisms: (1) The gut microbiota regulate the bladder microenvironment through metabolites (e.g., short-chain fatty acids); (2) Cross organ regulation mediated by neural pathways (such as vagus nerve) and shared receptors (e.g., FXR); (3) Immune factors affect the occurrence and development of bladder cancer through inflammatory reaction.

## 2 The gut-bladder axis: associated microbial communities

### 2.1 Gut microbiota, bladder microbiota, and their roles in bladder carcinogenesis

The role of microbiota in carcinogenesis was initially observed in animal studies, where gastrointestinal microbiota were shown to activate or produce carcinogens, which act locally in the gastrointestinal tract or remotely through urinary circulation or secretion to affect other organs (12). In previous research, the positive association between gut dysbiosis and colorectal cancer has been well established (13). Additionally, the gut microbiome has been demonstrated to influence cancer development and therapeutic responses (14). Gut dysbiosis contributes to the progression of multiple diseases, encompassing both gastrointestinal disorders and extraintestinal pathologies such as urological conditions (15, 16). A recent two-sample Mendelian randomization study confirmed that increased relative abundance of Bilophila in the gut was positively correlated with bladder cancer risk (10). Another genetic-level analysis exploring causal relationships between gut microbiota and urological cancers revealed that *Alistipes, Rikenellaceae, Lachnospiraceae UCG001*, and *Oscillibacter* in the gastrointestinal tract reduced bladder cancer risk, whereas *Eubacterium coprostanoligenes, Eubacterium fissicatena*, *Ruminococcus UCG013*, and *Thauera* were associated with elevated risk (12). As a critical disease in the urinary system, bladder cancer pathogenesis is linked not only to imbalances in the urinary tract microbiota but also to inflammatory and immune alterations driven by gut microbiota, which may initiate or modulate cancer progression (17). Although the gut-bladder axis remains incompletely defined, accumulating evidence suggests that specific gut microbial taxa may act as either pathogenic or protective contributors to bladder carcinogenesis.

Recent advancements in technology and research on human microbial communities have confirmed the presence of a microbiome within the urinary system (18–20). Previous studies suggest that urinary tract microbiota may contribute to carcinogenesis through chronic inflammation (21–23), potentially explaining observed microbial differences between bladder cancer patients and healthy individuals. Recent studies analyzing bacterial populations in bladder cancer patients (using urine, tissue, and paired tumor/non-tumor samples) revealed distinct microbial profiles. Comparative analysis of urinary microbiota between bladder cancer patients and healthy controls demonstrated significant elevations in *Fusobacterium, Campylobacter, Acinetobacter, Streptococcus, Bacteroides*, *Anaerococcus*, and *Micrococcus (* 21, 24–27 *)*, alongside marked reductions in *Lactobacillus* and *Firmicutes (* 25, 28, 29). These findings indicate that bladder cancer-associated microbiota are characterized by pathogenic enrichment and beneficial depletion. For instance, elevated *Fusobacterium* levels have been linked to chronic inflammation and toxin-mediated damage (30–32), while reduced *Lactobacillus* may foster an immunosuppressive microenvironment (25). Furthermore, microbial heterogeneity across sample types reflects distinct local (tumor) versus systemic ecological dynamics, necessitating future integration with multi-omics data for comprehensive elucidation.

### 2.2 Microbial crosstalk in the gut-bladder axis: pathogens, protectors, and functional roles

Alterations in gut microbiota exert profound influences on bladder microbial communities. Here, we focus on microbial taxa mediating gut-bladder interactions. The translocation of urinary pathogens from the gut to the bladder represents one of the most direct mechanisms linking UTIs to bladder carcinogenesis(**Table 1**). Urinary tract infections (UTIs), among the most prevalent bacterial infections globally, are predominantly caused by uropathogenic *Escherichia coli* (UPEC). The gut serves as the primary reservoir for UPEC and other uropathogens, functioning as a staging ground for their ascension into the bladder via proximal genitourinary mucosal niches (11). Similarly, *Streptococcus agalactiae* (Group B Streptococcus, GBS), a common commensal in the human gastrointestinal and genitourinary tracts, accounts for 2-3% of UTIs (33). Gut dysbiosis disrupts intestinal barrier integrity, allowing leakage of harmful solutes, pathogens, and toxins that trigger systemic inflammation and immune activation (33, 34). This mechanism extends the tumor-promoting effects of gut microbiota beyond the gastrointestinal tract to the bladder microenvironment.

**Table 1:** Microbial signatures and functional roles in the gut-bladder axis.

Classification	Associated microbiota	Gut alterations	Urinary tract alterations	Functional mechanisms
Pathogenic Bacteria	*Uropathogenic Escherichia coli* (UPEC)	Pathogen enrichment	Colonization-induced infection	LPS activates the TLR4/STAT3 pathway, driving pro-inflammatory responses.
	*Streptococcus* *Klebsiella*	Dysbiosis	Toxin-mediated epithelial damage	Virulence factors (e.g., proteases) are released, disrupting normal cellular integrity.
	*Eubacterium*	Dysregulated abundance	Elevated abundance in non-muscle-invasive BC	ERK1/2 phosphorylation pathway is activated, increasing MMP9 expression to enhance tumor invasiveness.
Beneficial Bacteria	*Lactobacillus* *Bifidobacterium*	Significantly reduced	Predominant in healthy urine	Antitumor immunity is enhanced via gut-tumor immune axis modulation by probiotic mixtures.
	*Prevotella*	Reduced abundance	Predominant in healthy female bladders	Potential protective role is hypothesized; mechanisms require further validation.
Under Investigation	*Bacteroides*	Statistically correlated	Markedly increased	Tumor progression may be bidirectionally regulated via FXR signaling pathway and cholesterol metabolism.

Gut microbiota may indirectly influence bladder microbiota and carcinogenesis through latent mechanisms(**Table 1**). A Chinese cohort study revealed significant gut dysbiosis in bladder cancer patients, marked by elevated *Streptococcus* abundance (35). A small pilot study further demonstrated enrichment of *Streptococcus* in urine samples from bladder cancer patients (36). One proposed mechanism involves microbial protease production, which interacts with epithelial surfaces. These enzymes act as extracellular virulence factors, degrading tissues, evading host defenses, and compromising physical barriers (36), thereby inducing inflammation and oxidative stress that promote carcinogenesis and recurrence. Within the Enterobacteriaceae family, *Klebsiella*, enriched in female hosts, produces toxins (e.g., colibactin) that directly drive bladder tumorigenesis (29). A Chinese metagenomic analysis identified dysregulated *Eubacterium* levels in the gut microbiota of bladder cancer patients (35). Another cohort study reported that upregulated urinary *Eubacterium* abundance alters extracellular matrix protein 1 (ECM1) in bladder tissue, enhancing matrix metalloproteinase 9 (MMP9) expression via the ERK1/2 phosphorylation pathway, ultimately facilitating bladder cancer progression (37). In BBN (N-butyl-N-(4-hydroxybutyl)nitrosamine)-treated mice, gut microbiota analysis revealed a significant increase in *Campylobacter* levels in bladder cancer models (38). This aligns with observations in human bladder cancer patients, where elevated *Campylobacter* abundance in bladder microbiota suggests its potential role as a key oncogenic pathogen (24).

Emerging evidence suggests that superficial bladder cancer therapies are influenced by gut and urinary tract microbiota (2, 4)(**Table 1**). Studies have revealed significant reductions in *Bifidobacterium, Lactobacillus*, and butyrate-producing bacteria in the gut microbiota of bladder cancer patients (35). Multiple studies confirm the positive role of probiotic mixtures containing *Lactobacillus* and *Bifidobacterium* in enhancing antitumor effects via the gut-tumor immune axis (39, 40). In a case-control study, He et al. observed decreased *Prevotella* abundance in the gut of bladder cancer patients, contrasting with its predominance in the bladder microbiota of healthy women (41). This association highlights *Prevotella* as a potential target for probiotic interventions in bladder cancer. A Mendelian randomization study implicated *Bacteroidetes* in the gut as a phylum linked to bladder cancer risk (12); while other work identified *Bacteroides* as a taxon significantly enriched in both controls and bladder cancer samples (42). Cholesterol metabolism-associated Bacteroides may serve as therapeutic or diagnostic targets for bladder cancer management.

## 3 Microbiota-metabolite-immune regulatory networks

### 3.1 Microbial metabolites: dual roles in inflammation and tumor modulation

**Figure 1:**
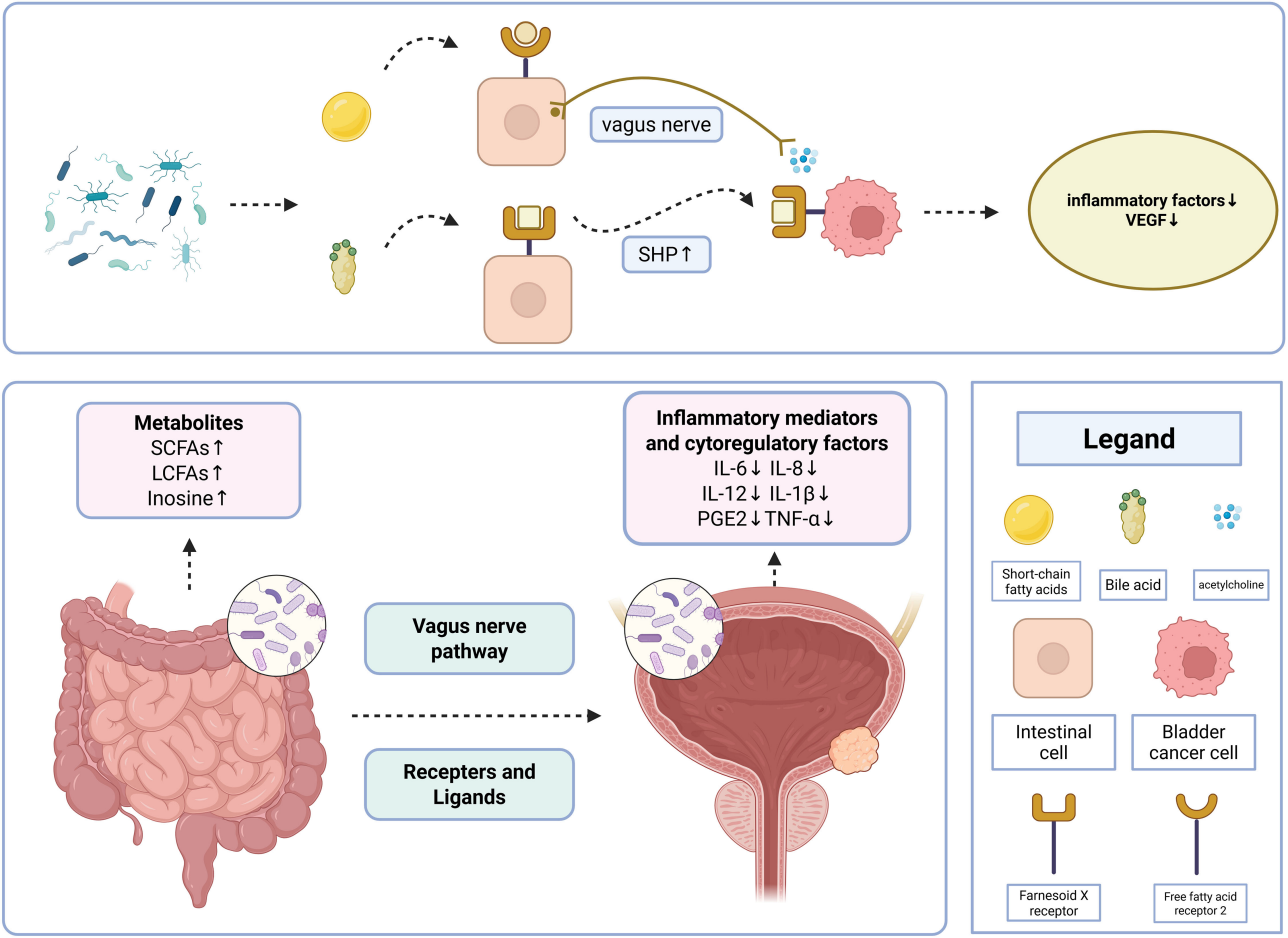
Interaction network of the gut-bladder axis.

The mechanism by which the gut microbiota regulates the development of bladder cancer can be explained by the introduction of a “microbiota-metabolite-immunity” network. Once absorbed through intestinal barriers, gut microbiota metabolites modulate inflammatory responses and the immune microenvironment, playing a key role in bladder cancer regulation (43)(**Figure 1**).

Short-chain fatty acids (SCFAs), including acetate, propionate, and butyrate, are key microbial metabolites predominantly localized in the proximal colon. By maintaining intestinal barrier integrity, promoting mucus production, and suppressing inflammation, SCFAs may directly inhibit cancer growth by preventing pro-tumorigenic epigenetic changes (44). Several taxa, including *E. faecalis* spp, *Ackermannia* spp, *Bradyrhizobium* spp, and *Fusobacterium harzianum*, which were reduced in the intestines of patients with recurrent urinary tract infections (rUTIs) in one study, were shown to be associated with the production of SCFAs, which exert anti-inflammatory effects in the intestinal tract through the promotion of intestinal barrier function and immunomodulation (45). Butyrate, propionate, and acetate all reduce IL-6 and IL-12 production in a dose-dependent manner, butyrate and propionate also decrease monocyte chemotactic protein-1 expression in LPS-treated monocytes, and the loss of SCFAs in dysbiosis of the gut microbiota moderates excessive immune activation, and inhibition of tumor growth may be suppressed (17). Not only that, SCFA can regulate histone deacetylase (HDAC), which affects cell adhesion, immune cell migration, cytokine production, chemotaxis and programmed cell death, influencing the development of UCB (46).

A comprehensive analysis found significant reductions in a number of long-chain fatty acids found in fecal samples from patients with bladder cancer, including 11Z-eicosenoic acid, pelargonic acid, vanillic acid, ricinoleic acid, oleic acid, and arachidic acid (35),and it can be hypothesized that low levels of LCFAs may be a contributing factor to the development of bladder cancer. Long-chain fatty acids (LCFAs) produced by gut microbiota can form a synergistic triad with the immune system to maintain homeostasis in the body through anti-inflammatory pathways, thus preventing the progression of inflammation-related cancers (47). In a study of *Escherichia coli Nissle 1917* (EcN), a probiotic used to treat a variety of intestinal disorders, in the gut, the concentration of 3-hydroxyoctadecadienoic acid (C18-3OH) was increased in EcN compared to the other E. coli strains tested, and the production of C18-3OH by the bacteria may be one of the mechanisms associated with the anti-inflammatory properties of the probiotic, LCFA-3OH production may be related to microbiota-host interactions that delay or ameliorate tumor progression (48).

Inosine plays a role in the therapeutic context in which *B. pseudomallei* enhances the efficacy of immune checkpoint blockade (ICB) therapies through the production of inosine. In the presence of exogenous IFN-γ, inosine strongly promotes the differentiation of TH1 naïve T cells and binds to the adenosine 2A receptor, leading to an inosine-A2 amp R-cAMP-PKA signaling cascade resulting in the phosphorylation of the transcription factor cAMP-responsive element-binding protein (CREB), which promotes Th1 differentiation and exerts its antitumor effect to inhibit the progression of bladder tumors and enhances the sensitivity to radiotherapy and chemotherapy. sensitivity to radiotherapy and chemotherapy (49).

### 3.2 Receptor-ligand axis: from microbial signals to cancer pathways

Receptors and ligands influence bladder cancer development directly or indirectly by mediating microbial signaling, metabolites, and immune modulation in the gut-bladder axis(**Figure 1**).

NR1H4, also known as Farnesoid X Receptor (FXR), acts as a nuclear receptor that is predominantly expressed in the liver and intestines and can be activated by binding to bile acids (BAs), and altering its expression can influence bladder carcinogenesis (50, 51). *Anaplasma* spp. in the intestinal tract as specific bacteria that produce bile acids (35, 52), can alter FXR expression to adjust metabolism. Excessive accumulation of bile acids in the intestinal lumen activates the FXR, leading to a marked upregulation of its downstream target, the small heterodimer partner (SHP), at the protein level (53). This FXR-SHP axis-driven signaling cascade propagates via systemic circulation, ultimately inducing overexpression of FXR in bladder tissues (53). Overexpression of FXR was highly correlated with bladder cancer progression, reducing the expression of integrin β1, integrin β3, p-FAK and p-MLC on the one hand, leading to the downregulation of the migration and adhesion ability of bladder cancer cells, and decreasing VEGF in the endothelial cells to inhibit angiogenic ability on the other hand, as well as enhancing the proteasomal degradation, leading to the downregulation of VEGFA, p-STAT3 and HIF1α and reduced angiogenesis to reach cancer inhibition (50).

TLR4, a receptor co-expressed in the gut and bladder, is critical for recognizing lipopolysaccharide (LPS) from Gram-negative bacteria and plays a role in maintaining intestinal barrier integrity (54, 55). In macrophages and the urinary mucosa, UPEC-derived LPS activates TLR4, leading to upregulated TLR4 signaling in urothelial cells. This signaling cascade induces the release of cytokines such as IL-6, IL-1β, IL-8, and PGE2 (17, 54). IL-6 activates STAT3 via the MAPK-STAT3 phosphorylation pathway, which supports cancer cell proliferation, survival, invasion, and metastasis (17, 54). STAT3 blockade in urothelial cells was shown to reduce bladder carcinogenesis and invasivity in murine models (56). IHC analysis demonstrated that STAT3 inhibition decreases TLR activation and suppresses proliferation in bladder cancer cell lines (57). Furthermore, TLR4 activation following UPEC infection in mice also triggers WNT-β-catenin signaling, which is closely linked to malignant transformation of bladder cells (58). β-catenin, a downstream target of WNT, is elevated in the bladder stroma post-infection. Genetic inhibition of β-catenin via a tamoxifen-inducible system in murine bladders was found to reduce stromal and urothelial proliferation during UTI (59).

### 3.3 Bladder-gut-brain axis: vagus nerve pathway as an intermediary

The gut-brain axis and neurological disorders have been increasingly well researched, but the number of studies addressing the neurological connections of the gut-bladder axis is limited. Leue et al. proposed the bladder-gut-brain axis (BGBA) hypothesis, which suggests that external/internal problems, such as psychiatric problems and infections, may contribute to bladder dysfunction (60). While the mechanisms are currently unclear, it is clear that urinary tract dysfunction frequently occurs in conjunction with gastrointestinal tract dysfunction, and that this coexisting urinary and gastrointestinal dysfunction may transmit danger signals from the body’s defense systems through the nervous system (60). BGBA is a useful framework for studying these interactions (61), and may be relevant to unraveling the mechanisms of bladder carcinogenesis(**Figure 1**).

Under the framework of the “microbiota-metabolite-immune” network, the vagus nerve acts analogously to a receptor network, activating immune mechanisms under the influence of microbial metabolites and regulating the bladder microenvironment. In a previous neuroanatomical tracer experiment in normal adult rats, it was found that a single vagal afferent could supply either the bladder or the colon or split in two and supply both organs, suggesting that the vagus nerve may be substantially involved in lower urinary tract and gastrointestinal tract function (62). Animal experiments have shown that vagotomy leads to an increase in the severity of inflammation following immune attack, making tumorigenesis significantly more likely (63, 64). And a 2024 study also confirmed the reduced parasympathetic activity observed in bladder cancer patients (65). Emerging studies have investigated the interplay between stress, psychological comorbidities, and female overactive bladder (OAB). A bidirectional brain-gut-bladder communication model has been proposed, implicating vagal dysregulation in bladder dysfunction pathogenesis (66). As a major pathway between the gut and the central nervous system, the vagus nerve may influence the bladder microenvironment through neuroimmune and metabolic pathways (67). Metabolites produced by gut microbiota (e.g., SCFAs) can bind to free fatty acid receptors FFAR2, FFAR3, and other G protein-coupled receptors (GPCRs) to induce secretion of intestinal hormones and indirectly activate the vagus nerve (61, 62, 68, 69). Inhibition of local IL-6/TNF-α release from systemic tissues, including the bladder, through cholinergic anti-inflammatory pathways suppresses local inflammation in the bladder and reduces the risk of cancer (70, 71). In a previous study, M2 muscarinic receptors suppressed cellular proliferation and migration in urothelial bladder cancer cells (72). Another report demonstrated significantly elevated expression of the nicotinic acetylcholine receptor α7 (nAChRα7) in nicotine-induced non-neoplastic bladder urothelial lesions (73). As a common neurotransmitter, acetylcholine may play a certain role in GBA-regulated bladder carcinogenesis. The role of the vagus nerve in the gut-bladder axis is unclear, and exploring vagus nerve stimulation (VNS) as an intervention to modulate gut microbiota and bladder cancer treatment is a very important potential future direction.

### 3.4 Multi-omics integration reveals dynamic host-microbiota crosstalk

The complexity of the gut-bladder axis (GBA) necessitates a systems-level approach to decipher the spatiotemporal interactions among microbial communities, host responses, and metabolic fluxes. While preceding sections describe isolated mechanisms (e.g., SCFA immunomodulation, FXR signaling), multi-omics integration (metagenomics, metabolomics, transcriptomics, proteomics) enables unbiased mapping of the “microbiota-metabolite-immunity” network. Metagenomic sequencing of fecal samples from bladder cancer patients revealed significant dysbiosis (e.g., reduced Bifidobacterium and Lactobacillus, enrichment of Streptococcus), with targeted metabolomics further identifying decreased fecal long-chain fatty acids (LCFAs) and increased cholesterol sulfate (35). KEGG functional annotation demonstrated significant downregulation of fatty acid biosynthesis and linoleic acid metabolism pathways in the gut microbiota of bladder cancer patients, consistent with reduced LCFAs (35). Notably, Clostridium sp. CAG_590 positively correlated with multiple LCFAs, while Bacteroides salyersiae showed a negative correlation, suggesting specific microbes modulate bladder cancer risk via metabolites (35). These metabolites cooperate with the immune system through anti-inflammatory pathways to maintain homeostasis and suppress inflammation-associated cancer (47). Conversely, spatial transcriptomics of bladder tumor tissue combined with 16S rRNA sequencing revealed that Fusobacterium enrichment in the tumor core correlated with sustained activation of the IL-6/STAT3 pathway, while Lactobacillus colonization at the tumor margin positively correlated with CD8+ T cell infiltration (27). This spatial heterogeneity accounts for the discrepancy between urine and tissue microbial profiles (25), and links microbiota to immunology. However, temporal dynamics remain poorly characterized due to limited longitudinal human data. Future studies must integrate metagenomics, metabolomics, and single-cell immunomics across gut/bladder niches to model real-time host-microbial metabolite fluxes and identify microenvironment-specific therapeutic targets.

As illustrated in the figure above, the gut-bladder axis regulates bladder cancer through a “microbiota-metabolite-immune” network. Gut microbiota-derived metabolites, including short-chain fatty acids (SCFAs), long-chain fatty acids (LCFAs), and adenosine, act on bladder cancer-specific targets via receptor-mediated pathways or vagus nerve signaling, inducing changes in immune factors such as IL-6 and IL-8, which play critical roles in suppressing bladder cancer initiation and progression. The two most canonical pathways are as follows:

#### 3.4.1 SCFA-FFAR2-cholinergic anti-inflammatory pathway

Gut microbiota-produced SCFAs bind to free fatty acid receptor 2 (FFAR2), activating the vagus nerve-mediated cholinergic anti-inflammatory pathway. This pathway inhibits the release of IL-6 and TNF-α in bladder cells.

#### 3.4.2 Bile acid-FXR-SHP axis

Gut microbiota-metabolized bile acids activate the Farnesoid X receptor (FXR), a receptor shared by intestinal and bladder tissues. This enhances the protein expression of the downstream target small heterodimer partner (SHP), leading to FXR overexpression in bladder cells. This process remodels the tumor microenvironment, reduces vascular endothelial growth factor (VEGF) secretion, and suppresses bladder cancer metastasis.

## 4 A new treatment for bladder cancer based on microecological theory

### 4.1 Gut flora enhances efficacy of immune checkpoint inhibitors

Recent studies have shown that intestinal microbiota play a decisive role in the treatment of immune checkpoint inhibitors, and because of their ability to activate the immune system, research is underway into whether bacteria can be used as an immunotherapeutic tool (74). Single bacterial colonization of *Dictyostelium parvum* in the intestine combined with immunosuppressive agents significantly increased the intratumoral expression of CD4+ and CD8+ cells, suggesting that *Dictyostelium parvum* in combination with immunotherapy with ICIs may enhance the antitumor effect of α-PD-1mAb in tumor-bearing mice by increasing the infiltration of some immune cells (75). Accumulating evidence demonstrates that the gut microbiome modulates the response to immune checkpoint inhibitors (ICIs) in cancer patients. Multiple groups have independently demonstrated a link between the gut microbiome and immunotherapy response in cancer patients. A significant association was observed between commensal microbial composition and clinical response. Bacterial species that were more abundant in responders included *Bifidobacterium longum, Collinsella aerofaciens*, and *Enterococcus faecium.* Reconstructing germ-free mice with fecal microbiota from responding patients improved tumor control, enhanced T-cell responses, and increased the efficacy of anti-PD-L1 therapy. The results suggest that the commensal microbiome may have a mechanistic impact on antitumor immunity in human cancer patients (76).

### 4.2 Probiotics reverse chemotherapy resistance

Chemotherapy, the mainstay of treatment for inoperable cancers, suffers from a number of drawbacks, such as insufficient drug concentrations in tumors, the occurrence of systemic toxicity (hematological, gastrointestinal, alopecia, cardiac, and dermatological toxicity) in many types of cancers, and the almost inevitable induction of drug resistance (77). Clinical trials and reviews of human, animal and *in vitro* studies have shown that probiotic interventions can exert anti-tumor effects through cancer cell apoptosis and immunomodulation, anti-angiogenic and anti-metastatic activities (40). The use of probiotics was found to have a beneficial effect on the prognostic outcome of chemotherapy in one study. The combination of probiotics with gemcitabine and cisplatin increased the antitumor effect in terms of decreasing the size of the implanted tumors compared to the combination or individual administration (78). In addition, probiotic supplementation activated antigen-presenting processes, including increased recruitment of cytotoxic T cells, which could explain the enhanced antitumor effects (79).

### 4.3 The role of gut microbiota in neoadjuvant combination therapy for bladder cancer

In recent years, neoadjuvant combination therapies (such as chemotherapy combined with immunotherapy, or antibody-drug conjugates combined with immunotherapy) have demonstrated high pathological response rates and survival benefits in muscle-invasive bladder cancer (80, 81). However, patient responses to treatment exhibit significant individual heterogeneity, in which the gut microbiota may play a crucial role. Multiple studies indicate that the gut microbiota influences the efficacy of cancer therapies by modulating systemic immune responses. In melanoma and non-small cell lung cancer, specific gut microbial taxa (e.g., Bifidobacterium, Akkermansia) correlate with enhanced efficacy of immune checkpoint inhibitors (76, 82). Furthermore, antibiotic-induced gut dysbiosis is associated with reduced effectiveness of immunotherapy (83). In the context of bladder cancer, the gut microbiota composition of patients differs significantly from that of healthy individuals, characterized by a reduction in short-chain fatty acid-producing bacteria (35). These microbial alterations may contribute to an immunosuppressive microenvironment, thereby potentially affecting the efficacy of neoadjuvant therapies. Although current research on the dynamic changes of gut microbiota during neoadjuvant combination therapy remains limited, animal models have validated that gut microbiota enhances the antitumor effects of chemotherapeutic agents (40). Modulating the gut microbiota holds promise for application in neoadjuvant combination therapy for bladder cancer to improve pathological complete response rates and create opportunities for bladder-preserving treatment.

### 4.4 Bacterial recombination and clinical translation

Gut microbiota have brought about a sea change in the way cancer is treated, and engineered bacteria targeting and regulating bladder cancer is an important future endeavor. The latest study inserted IL-2 and TRAIL genes into the genome of attenuated *Salmonella* strain SL3261, which was then inoculated into bladder cancer lineage cells in mice, and successfully promoted apoptosis in mouse bladder cancer M49 cells (84). Targeting microbiota-derived metabolites has emerged as a novel strategy to reshape bladder cancer treatment paradigms. In one study, sodium butyrate, a short-chain fatty acid derivative, was shown to inhibit cell growth and induce apoptosis in bladder cancer cell lines through the miR-139–5p/Bmi-1 axis. Autophagy and ROS overproduction were triggered via AMPK/mTOR pathway activation, which was linked to cytotoxic effects (85). The mechanism of the gut-bladder axis is based on the formation of a “microbiota-metabolite-immunity” regulatory axis of microbial metabolites (SCFAs/LCFAs) via receptors (FXR/TLR4) and neural pathways (vagus nerve) that influence bladder carcinogenesis. Experimental studies have demonstrated that the FXR agonist GW4064 reduces colony formation and effectively inhibits the migration and invasion of human bladder cancer cells (86). On the other hand, the latest breakthroughs in synthetic biology, particularly the CRISPR-Cas system, offer promising solutions for overcoming the limitations of strain specificity and dosage in microbial therapy (87). Recently, engineered butyrate-producing *Bacillus subtilis strain BsS-RS0655* was constructed based on the CRISPR-Cas9 genome editing system (88), as CRISPR-based genome editing will enable precise genetic modifications to probiotic strains. Recent studies have utilized pH-responsive polyserotonin (PST) to encapsulate *Salmonella*, addressing the issues of systemic clearance and toxicity associated with traditional bacterial therapies (89). Tumor cell membrane (TCM) has been validated as a cancer vaccine carrier with significant potential for immunotherapy (90), and its combination with engineered bacteria represents a novel concept for bladder cancer treatment. For instance, designing synthetically engineered probiotics (such as *Lactobacillus* strains expressing SCFAs synthases) delivered orally or locally to the bladder could potentially precisely modulate the metabolic functions of the gut/bladder microbiota, achieving gut-bladder axis-targeted therapy.

## 5 Targeted regulation of microbiota and clinical translation

### 5.1 The gut-bladder axis and other diseases

The gut and bladder, as the anatomical endpoints of the gut-bladder axis, hold significant research value in understanding the link between colorectal cancer (CRC) and bladder cancer (BC). Gut dysbiosis, a shared risk factor for both malignancies, has been definitively linked to CRC through microbial taxa such as *Fusobacterium nucleatum, Bacteroides fragilis*, and *enteropathogenic Escherichia coli* (EPEC) (91–93). In BC, uropathogenic Escherichia coli (UPEC) and Streptococcus species are more specifically implicated. Organ-specific mechanisms are proposed: direct microbial-epithelial interactions drive CRC pathogenesis, whereas bladder carcinogenesis is mediated indirectly via metabolites (e.g., lipopolysaccharide) or neural signaling pathways. The gut-bladder axis represents a unique cross-organ regulatory mechanism in BC. Investigation of reverse signaling (bladder-to-gut) may open therapeutic avenues for CRC.

A two-sample Mendelian randomization study established causal associations between gut microbiota composition and interstitial cystitis (94). NLRP3 inflammasomes, produced by gut microbiota, have been identified as mediators of urological pathologies, promoting bladder inflammation and fibrosis through IL-1β and IL-18 activation (95). The gut-bladder axis is hypothesized to reshape diagnostic and therapeutic paradigms for bladder disorders. Defining its precise role in disease pathogenesis remains a critical unmet challenge.

### 5.2 Microbial associations between schistosomiasis and bladder cancer

Beyond specific microbial taxa, schistosome-induced dysbiosis in both gut and urinary microbiota has been strongly linked to bladder carcinogenesis. Schistosomiasis-endemic regions like Egypt and sub-Saharan Africa show markedly elevated bladder cancer incidence, with squamous cell carcinoma (SCC) predominating (50-80% of cases) - substantially exceeding the global average (<5%) (96). Although exact mechanisms remain incompletely understood, urogenital schistosomiasis alters urinary tract microecology. Chronic urothelial inflammation and mechanical damage from egg deposition in bladder walls are recognized drivers of carcinogenesis (97–99). Recent murine studies demonstrate significant enrichment of *Verrucomicrobia phylum* members (particularly *Akkermansia muciniphila*) and *Lactobacillus* in Schistosoma mansoni-infected gut microbiomes (100). This evidence suggests schistosomal carcinogenesis may involve cross-talk between gut/urinary microbiota, positioning infection-related dysbiosis as a potential risk biomarker. Future investigations combining metagenomic profiling with host immune analyses should be prioritized to elucidate specific molecular targets in schistosome-microbiota interactions.

### 5.3 Gut-bladder axis and xenobiotic metabolism

Exposure to environmental pollutants and the composition of the human microbiome are critical predisposing factors for tumor development (101). Recent studies highlight the potential of human gut bacteria to metabolize chemically diverse compounds, such as pharmaceuticals, with their metabolites distributed to distal tissues (102). A recent murine study demonstrated that gut microbiota in humanized mice significantly influenced bladder carcinogenesis induced by N-butyl-N-(4-hydroxybutyl)-nitrosamine (BBN) exposure (103). Specifically, gut microbiota oxidizes BBN into N-butyl-N-(3-carboxypropyl)-nitrosamine (BCPN), which induces tumorigenesis via DNA adduct formation in the urothelium (104). Flavanols, secondary plant metabolites found in various common foods, are metabolized by gut microbes into compounds (e.g., valerolactones, phenylalkyl acids, and hippuric acids) excreted via urine. Notably, these microbial metabolites exhibit a positive correlation with reduced bladder cancer proliferation *in vitro*, suggesting that gut microbiota may mediate the chemopreventive effects of flavanol intake on urothelial cells through microbial-derived metabolites (105). These findings not only confirm that gut microbiota regulate bladder carcinogenesis via endogenous metabolites but also reveal their role in metabolizing xenobiotics to influence cancer progression, offering novel strategies for bladder cancer prevention and therapy.

### 5.4 Limitations

Significant variations in gut and bladder microbiota composition across regions, sexes, and age groups have been observed, raising concerns about the generalizability of current findings. For instance, while *Bacteroides* has been reported to exert antitumor effects via bile acid pathways, contradictory findings were noted in a study showing elevated Bacteroides abundance in the bladder microbiota of BC patients. Current studies are predominantly focused on isolated mechanisms (e.g., specific taxa or metabolites), whereas systemic integration of the “microbiota-metabolite-immune-neural” network remains understudied. For example, the specific signaling pathways through which SCFAs suppress bladder inflammation via the vagus nerve remain incompletely elucidated. Most experimental models (e.g., BBN-induced murine bladder cancer) fail to fully recapitulate human tumor microenvironments. Translational challenges persist: probiotic interventions have shown reduced BC recurrence rates in some trials, but efficacy is influenced by strain specificity, dosage, and host immune status, with no standardized protocols established. Additionally, the colonization stability and long-term safety of synthetic biology-engineered bacterial strains have not been validated. The core challenge in current gut-bladder axis research lies in the limited generalizability of conclusions due to geographic, gender, and age-related heterogeneity in microbiota composition. Multicenter stratified cohorts become crucial for addressing population heterogeneity121 (96). Integrating patient-derived organoid models with synthetic biology-engineered bacterial technology can advance personalized modulation of the “microbiota-metabolite-immunity” axis. One study utilized bladder organoids to validate the anti-proliferative effects of flavanol microbial metabolites in urine109 (105). Organoids, as essential tools for simulating tumor microenvironments, will facilitate personalized treatment customization. For instance, combination regimens of probiotics and immune checkpoint inhibitors could be tailored on models according to intestinal permeability and microbial profiles, with targeted delivery achieved through CRISPR-edited engineered bacteria.

## 6 Conclusion

The gut-bladder axis has emerged as a key interface linking gut ecological dysregulation, metabolic disorders and neuroimmune dysregulation to bladder carcinogenesis. A growing body of evidence emphasizes that imbalances in the gut microbiota, characterized by the enrichment of pathogenic organisms and the absence of beneficial taxa, disrupt systemic immune homeostasis and promote a pro-inflammatory microenvironment that favors bladder cancer progression. Microbial metabolites, such as SCFAs and LCFAs, are key mediators in the regulation of immune responses, epigenetic modifications, and cellular signaling pathways (e.g., HDAC inhibition, FXR activation, and TLR4/STAT3 signaling). Notably, the vagus nerve-mediated bladder-bladder axis further integrates neural and immune crosstalk, highlighting the multifaceted nature of bladder cancer pathogenesis.

Emerging therapeutic strategies targeting the gut-bladder axis, such as probiotic interventions, engineered microbiota, and immunomodulatory metabolites, show promise in enhancing chemosensitivity and immune checkpoint inhibitor efficacy. However, clinical translation remains hampered by challenges such as inter-individual microbial variation, limited mechanistic understanding of neuroimmune interactions, and the complexity of host microbial metabolic networks. Building on current advancements and challenges, future research should prioritize the development of probiotics capable of precisely modulating microbiota-metabolite-immune signaling pathways. These engineered probiotics could enhance the anti-tumor immune microenvironment to suppress metastasis or alleviate inflammation, thereby improving chemotherapy efficacy. Concurrently, integrating patient-specific genomic, metabolic, and immunological data with organoid models derived from patient cells could simulate gut-bladder interactions, enabling the design of personalized microbiota-targeted therapies to address interindividual variability in treatment outcomes. These strategies demand multidisciplinary collaboration and technological innovation to overcome existing barriers, ultimately advancing precision prevention and treatment for bladder cancer.
